# Environmental Drivers of Benthic Flux Variation and Ecosystem Functioning in Salish Sea and Northeast Pacific Sediments

**DOI:** 10.1371/journal.pone.0151110

**Published:** 2016-03-04

**Authors:** Rénald Belley, Paul V. R. Snelgrove, Philippe Archambault, S. Kim Juniper

**Affiliations:** 1 Departments of Ocean Sciences and Biology, Memorial University of Newfoundland, St. John’s, Newfoundland and Labrador, Canada; 2 Institut des sciences de la mer de Rimouski, Université du Québec à Rimouski, Rimouski, Québec, Canada; 3 School of Earth and Ocean Sciences, University of Victoria, Victoria, British Columbia, Canada; Auckland University of Technology, NEW ZEALAND

## Abstract

The upwelling of deep waters from the oxygen minimum zone in the Northeast Pacific from the continental slope to the shelf and into the Salish Sea during spring and summer offers a unique opportunity to study ecosystem functioning in the form of benthic fluxes along natural gradients. Using the ROV ROPOS we collected sediment cores from 10 sites in May and July 2011, and September 2013 to perform shipboard incubations and flux measurements. Specifically, we measured benthic fluxes of oxygen and nutrients to evaluate potential environmental drivers of benthic flux variation and ecosystem functioning along natural gradients of temperature and bottom water dissolved oxygen concentrations. The range of temperature and dissolved oxygen encountered across our study sites allowed us to apply a suite of multivariate analyses rarely used in flux studies to identify bottom water temperature as the primary environmental driver of benthic flux variation and organic matter remineralization. Redundancy analysis revealed that bottom water characteristics (temperature and dissolved oxygen), quality of organic matter (chl *a*:phaeo and C:N ratios) and sediment characteristics (mean grain size and porosity) explained 51.5% of benthic flux variation. Multivariate analyses identified significant spatial and temporal variation in benthic fluxes, demonstrating key differences between the Northeast Pacific and Salish Sea. Moreover, Northeast Pacific slope fluxes were generally lower than shelf fluxes. Spatial and temporal variation in benthic fluxes in the Salish Sea were driven primarily by differences in temperature and quality of organic matter on the seafloor following phytoplankton blooms. These results demonstrate the utility of multivariate approaches in differentiating among potential drivers of seafloor ecosystem functioning, and indicate that current and future predictive models of organic matter remineralization and ecosystem functioning of soft-muddy shelf and slope seafloor habitats should consider bottom water temperature variation. Bottom temperature has important implications for estimates of seasonal and spatial benthic flux variation, benthic–pelagic coupling, and impacts of predicted ocean warming at high latitudes.

## Introduction

Marine carbon, nitrogen, and phosphate cycles are linked through their fixation by phytoplankton in surface waters and their remineralization in the water column and on the seafloor [1]. The decomposition of organic matter on the seafloor and resulting early diagenetic reactions at the sediment–water interface significantly impact nutrient composition within the water column and associated ecosystem processes, releasing 25–80% of the essential nutrients (e.g., N, P, and Si) that fuel primary production in the photic zone of shallow water (<50 m) systems [2]. This co-dependence of processes between the water column and sediments, which varies with water depth [3], defines benthic–pelagic coupling [4].

On the continental shelf, close benthic–pelagic coupling typically occurs over time scales of days. The benthic community generally responds rapidly to increased particulate organic carbon (POC) flux to the seafloor, resulting in measurable increases in benthic respiration [5]. This response to increased food supply may be rapid and limited in duration when food pulses to the seafloor vary seasonally, as seen in the Arctic [6, 7] and in some deep-sea regions [8–10]. If organic matter (OM) sinks through several hundred meters in the water column before reaching the seafloor, the lag between peak surface primary production and settlement of associated OM particles on the seafloor may span weeks [11]. In addition to an increase in oxygen uptake, several studies correlate POC flux reaching the seafloor with increased abundance and activity in bacteria, foraminifera, and meiofauna [9, 12].

Ecosystem functioning of benthic habitats, such as organic matter remineralization, depends strongly on biological (e.g., faunal activities and diversity) and environmental (e.g., particulate organic matter flux to the seafloor) factors [13, 14]. Although many previous studies investigated the effects of biological drivers on ecosystem processes and functions under controlled conditions (see reviews of Duffy [15], Hooper et al. [16], Stachowicz et al. [17]), the few studies that also addressed one or more abiotic drivers have shown that environmental variables also play a key role in controlling ecosystem functioning [13, 18–20]. Among them, most studies identify bottom water temperature [21–23], dissolved oxygen concentration [22] and POC flux to the seafloor [24, 25] as the most important environmental drivers of benthic fluxes and organic matter remineralization. In the field, *in situ* studies that utilize natural variation such as those that occur along environmental gradients could potentially highlight the main environmental drivers of benthic fluxes, organic matter remineralization and ecosystem functioning in natural systems [26].

Nutrient regeneration, a central contribution of marine benthic habitats to ecosystem functioning, can be quantified by measuring oxygen and nutrient fluxes at the sediment–water interface [27]. Many benthic flux studies use sediment core incubations which, despite some artefacts [2], produce reliable estimates of benthic flux in water depths <1000 m (e.g., oxygen measurements [28]) and therefore provide a valuable tool for studying spatial and temporal variation in benthic fluxes.

The decomposition of organic matter at the sediment surface releases or takes up multiple dissolved nutrients such as ammonium, nitrate, nitrite, silicate, and phosphate, but the vast majority of published studies used oxygen uptake as a proxy for organic matter remineralization. Even so, oxygen uptake may not necessarily be the best variable to evaluate seafloor organic matter oxidation [29], and a recent study in the Beaufort Sea showed that oxygen flux poorly represented other nutrient dynamics [30]. Here, we examine multiple flux measures of OM constituents and determine the effectiveness of multivariate analyses in discerning among potential drivers of ecosystem function and providing a more global understanding of benthic remineralization patterns and drivers.

Our study focuses specifically on two contrasting seabed environments in the Northeast Pacific. The Salish Sea, a semi-enclosed inland sea between Vancouver Island and mainland British Columbia, Canada, was chosen because past studies indicated strong seasonality in temperature, dissolved oxygen [31], and primary productivity [32, 33]. In contrast, the British Columbia continental slope offers a deep seafloor study site within an oxygen minimum zone (OMZ, dissolved O_2_ < 0.5 mL L^-1^); the OMZ affects the Northeast Pacific continental shelf and Salish Sea bottom waters to different degrees (see Sect. 2.2). The comparison of two very different environments offers an opportunity to study the drivers of benthic flux along natural gradients of bottom water dissolved oxygen concentration and temperature. Furthermore, recent declines in dissolved oxygen concentrations in both study areas add to the importance of understanding the role of dissolved oxygen in determining benthic fluxes.

The bottom waters off Vancouver Island on the British Columbia continental shelf and slope experienced declining oxygen concentrations from the early 1980s to 2011, declining at rates of 0.019 to 0.025 mL L^-1^ y^-1^ [34]. This phenomenon partly explains the observed 0.02 to 0.03 mL L^-1^ y^-1^ oxygen decrease in the bottom waters of the Salish Sea (Strait of Georgia) between 1970 and 2006 [31, 33]. The oxygen concentration in the deep Strait of Georgia now reaches a minimum of ~ 2 mL L^-1^ [35]. Ongoing climate warming associated with human activities [36] could further change seawater properties (e.g., increase temperature, decrease dissolved oxygen and pH) and thereby affect biogeochemical fluxes in the Salish Sea [33].

The main objective of this study was to apply a suite of multivariate analyses widely used by ecologists to measurements of ecosystem functioning related to sediment geochemistry. Specifically, we examined benthic oxygen and nutrient fluxes in order to determine environmental drivers of spatial and temporal variation in organic matter remineralization. We addressed our objective by exploring the following research questions: i) do regional flux differences exist between the Salish Sea and NE Pacific, ii) do Salish Sea sites vary spatially and temporally, iii) do NE Pacific sites vary spatially in remineralization and nutrient flux, and iv) which environmental variables drive benthic fluxes and remineralization at our study sites?

## Materials and Methods

### Field sampling

**Fig 1:**
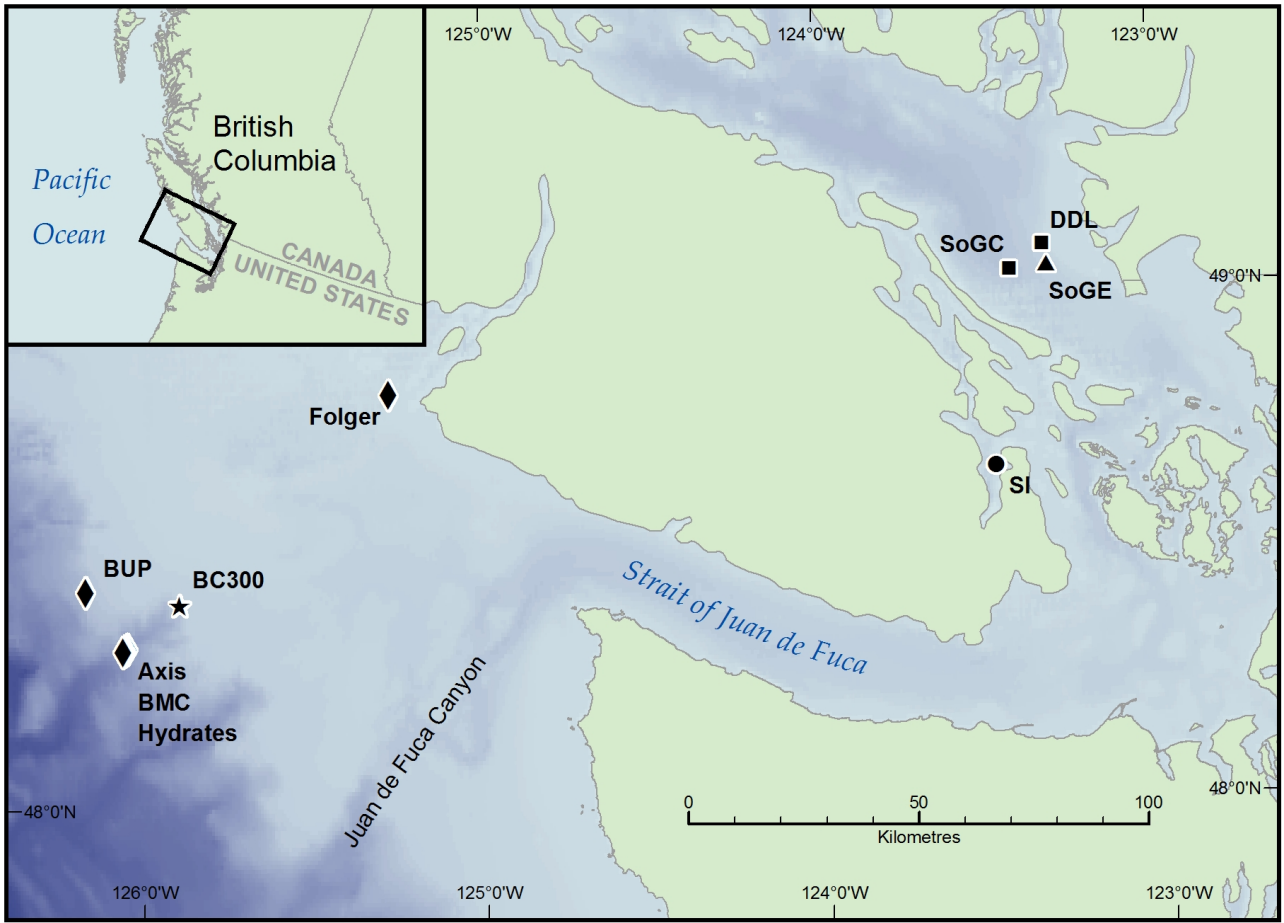
Map of stations sampled in the Salish Sea and the North East Pacific in May/July 2011 and September 2013. Label symbols indicate sampling dates. 1) Squares: DDL and SoGC were sampled in May and July 2011; 2) Triangle: SoGE was sampled in May 2011 and September 2013; 3) Circle: SI was sampled in July 2011 and September 2013; 4) Diamonds: Axis, BMC, Hydrates, BUP and Folger were sampled in July 2011; 5) Star: BC300 was sampled in September 2013. Bathymetry data based on the GEBCO_2014 Grid, version 20150318, www.gebco.net.

Sampling targeted locations near the VENUS Observatory nodes in the Salish Sea and some NEPTUNE Observatory nodes on the continental shelf and slope off Vancouver Island (Fig 1). These observatories are operated by Ocean Networks Canada (www.oceannetworks.ca). We collected push core sediments using the Remotely Operated Vehicle (ROV) ROPOS (www.ropos.com) on board the Canadian Coast Guard Ship John P. Tully (May 7–14, 2011), and the Research Vessels Thomas G. Thompson (June 30–July 3, 2011) and Falkor (September 6–18, 2013). We sampled the VENUS Strait of Georgia Central (SoGC) and the Delta Dynamic Laboratory (DDL) sites in May and July 2011, the Strait of Georgia East (SoGE) in May 2011 and September 2013, and Saanich Inlet (SI) in July 2011 and September 2013 (Table 1). NEPTUNE sites were sampled in July 2011 except for BC300, which we sampled in September 2013. The ROV collected 3–10 push-cores at each site (i.d. = 6.7 cm, L = 35.6 cm) at random locations. One core per site served to determine prokaryotic cell abundance and sediment properties, and the remaining cores were used for incubations to measure fluxes. A SBE 19plus V2 CTD mounted on the ROV recorded near-bottom dissolved oxygen (DO), temperature, and salinity. No specific permissions were required for these locations/activities and field studies did not involve endangered or protected species.

**Table 1:** Station names, sampling dates, number of incubations performed, locations and environmental variables measured. Bottom DO = Bottom water dissolved oxygen concentration; OPD = Oxygen penetration depth; Chl *a*:Phaeo = Chlorophyll-*a* to phaeopigments ratio; C:N = Carbon-to-nitrogen ratio; MGS = Sediment mean grain size.

Station	Date	Inc (#)	Lat (N)	Long (W)	Depth (m)	Temp (°C)	Bottom DO (mL L^-1^)	OPD (mm)	Chl *a*: Phaeo	C:N	Porosity (%)	MGS (μm)	Prokaryotic abundance (# cells g^-1^)
SI	07–2011	3	48°39.25	123°29.20	97	8.72	1.51	4.7	0.23	8.42	66.28	78.62	3.45E+08
SI	09–2013	4	48°39.25	123°29.17	97	9.24	0.97	3.7	0.23	10.01	73.48	87.76	7.66E+07
SoGE	05–2011	4	49°02.56	123°19.15	173	8.25	4.88	13.0	0.22	9.51	64.31	87.29	1.01E+08
SoGE	09–2013	4	49°02.55	123°18.97	167	9.65	2.42	5.8	0.18	34.89	64.40	112.86	7.57E+07
SoGC	05–2011	4	49°02.16	123°25.68	305	9.14	2.59	9.0	0.18	8.64	64.31	38.28	1.60E+08
SoGC	07–2011	3	49°02.42	123°25.51	301	8.63	2.86	12.0	0.21	8.77	83.64	27.30	9.07E+07
DDL	05–2011	2	49°05.05	123°19.76	109	8.27	4.95	10.0	0.37	11.66	65.52	75.08	1.64E+08
DDL	07–2011	3	49°05.05	123°19.75	107	8.91	3.23	14.7	0.59	16.97	60.79	95.66	1.48E+08
Axis	07–2011	3	48°19.01	126°03.03	987	3.87	0.19	8.0	0.16	10.56	81.10	46.86	9.74E+07
Hydrates	07–2011	3	48°18.71	126°03.95	868	4.26	0.20	7.3	0.18	9.66	80.18	33.12	9.09E+07
BMC	07–2011	3	48°18.88	126°03.49	896	4.22	0.19	7.3	0.19	9.34	85.43	32.21	1.56E+08
BUP	07–2011	3	48°25.66	126°10.48	397	5.56	0.71	7.5	0.17	14.17	51.05	124.53	1.07E+08
Folger	07–2011	3	48°48.83	125°16.85	96	7.75	2.01	11.5	0.18	8.89	81.69	44.26	4.66E+08
BC300	09–2013	3	48°24.17	125°53.89	298	6.62	1.06	5.6	0.14	19.44	60.29	164.82	8.54E+07

### Study area hydrographic description

Ocean circulation around our study area results from complex interactions between multiple currents, eddies, and water masses, as well as tides and seasonal wind patterns. An oxygen minimum zone (OMZ) at intermediate depth (400–1000 m) [37] adds complexity to continental slope waters along the west coast of North America, including our Barkley Canyon mid-slope sites [38]. Summertime northerly winds upwell this low-oxygen deep water to the continental shelf [34, 39, 40]. Estuarine circulation, driven by freshwater from the Fraser River at the north in the Strait of Georgia, facilitates subsurface movement of hypoxic water into the Salish Sea through the Strait of Juan de Fuca. Re-oxygenation of this deep water can occur during transport over the sill at the tidal passages of Haro Strait, replenishing oxygen in Strait of Georgia deep waters in late spring and late summer [33, 35, 41]. Finally, during fall, these same deep waters enter Saanich Inlet, a seasonally hypoxic fjord, by flowing over the sill from Haro Strait and replenishing oxygen-depleted bottom waters in the basin of the fjord [42].

### Incubations

At each sampling site we incubated 2–4 sediment cores by capping the bottom of each core and, where necessary, topping up core tubes with bottom water collected from the same site by the ROV suction sampler. Sediment cores were incubated in a dark cold room at *in situ* temperatures (4–9°C), in May and July 2011, and in a modified chest freezer in September 2013. The high sampling intensity, tight dive schedule, and limited number of incubations that could be run simultaneously meant that core acclimation times to allow sediment particles in suspension in the overlying water to settle back to the sediment surface varied from 4–24 hours, which is within the normal range of acclimation time reported in the literature [30, 43, 44]. Before the end of the acclimation period and just before the onset of incubations, we simultaneously aerated the overlying water in each core for a minimum of one hour using aquarium air pumps to avoid suboxic conditions during incubations. At the beginning of the incubations, we hermetically sealed cores with caps equipped with magnetic stirrers and gas-tight sampling ports. This system constantly stirred and homogenised the overlying water gently without resuspending surface sediments. Incubations ran for 12–48 hours until 15–30% of available oxygen was consumed and the volume of the overlying water was 444 ± 90 mL (mean ± SE).

### Oxygen uptake

We measured oxygen consumption periodically (4–8 hours intervals) using a 500-**μ**m oxygen microsensor (Unisense, Aarhus, Denmark) inserted through a small resealable hole on the top of the cap in May and July 2011, and with a non-invasive optical oxygen meter used in conjunction with oxygen optode patches (Fibox 4, PreSens, Regensburg, Germany) in September 2013. We then determined oxygen uptake from the slope of the linear regression of oxygen concentration versus time of incubations after correction for the oxygen concentration in the replacement water.

### Nutrient fluxes

At the beginning, midpoint, and end of the incubations we collected water samples with 60-mL, acid-rinsed plastic syringes, except in the SI, SoGC, and DDL incubations in July 2011 where high oxygen consumption limited water sampling to the beginning and end of the incubations as a result of the shortened incubation period (12 hours). We immediately replaced withdrawn water with an equivalent volume of bottom water of known oxygen and nutrient concentrations. Syringes and sample containers were initially rinsed with ~5 mL of water sample. At each sampling time we collected and stored two 25-mL water samples in acid-rinsed twist-cap 30-mL HDPE bottles. Upon collection, water samples were immediately placed in an upright position at -20°C until analysed. Given the numerous nutrients analysed, the risk of contamination, and the absence of suspended particles in water samples, we followed Aminot & Chaussepied [45] and chose not to filter water samples prior to storage. In the few instances where suspended particles were present in water samples, we allowed particles to settle and excluded them from the analysis [46]. We determined the concentrations of nutrients (NH_4_^+^, NO_3_^-^, NO_2_^-^, Si(OH)_4_ & PO_4_^3-^) in the water samples using a Technicon Segmented Flow AutoAnalyzer II, following the method recommended by Technicon Industrial Systems [47–49] with the exception of ammonia (hereafter referred as ammonium) analysis, which followed Kerouel & Aminot [50]. Nutrient fluxes were determined from the slope of the linear regression of nutrient concentrations versus time of incubations after correction for the solute concentration in replacement water.

### Effect of overlying water air bubbling on benthic flux rates

We added a complementary experiment at BC300 in September 2013 to determine whether air bubbling before the onset of the incubations affected benthic flux rate measurements. For this experiment, we processed four cores exactly as described above but added a second treatment in which we gently topped up five additional cores with bottom water collected *in situ* and quickly sealed them to maintain oxygen concentrations as close to *in situ* condition as possible but without bubbling. We held replacement water to exchange with withdrawn water in a sealed container equipped with an optical oxygen patch (see above) to measure DO in replacement water. We measured overlying water DO prior to incubations, and performed O_2_ uptake and nutrient analyses as described above.

### Oxygen penetration depth (OPD)

Immediately after recovery of the ROV we profiled oxygen concentrations as a function of depth in the sediment for one sediment core from each site. In each core, we performed three replicate profiles with Unisense oxygen microsensors (500 μm and 250 μm tip sizes in 2011 and 2013, respectively) in vertical increments of 1000 μm and 500 μm in 2011 and 2013, respectively. We defined the oxygen penetration depth (OPD) in the sediment as the mean depth where oxygen concentration decreased below the suboxic level of 5 μmol L^-1^ [51].

### Prokaryotic cells

To sample sediment prokaryotes we subcored the sediment cores with a cut off 10-mL sterile plastic syringe at depths of 0–2, 2–5 and 5–10 cm. We placed 1 mL of sediment from each depth in a 20-mL scintillation vial containing 4 mL of a filtered-sterilized 2% seawater-formalin solution. Samples were frozen at -20°C until analysis. Sediment prokaryote abundance and biomass were determined following Danovaro [52].

### Sediment properties

To characterize sediment properties we sectioned the upper 2-cm layer of sediment from one sediment core using inert plastic spatulas. Each sediment layer was carefully placed in a Whirl-Pak bag and stored at -20°C until analysed. Total organic matter (TOM) was determined by ignition loss, and water content as the difference between the wet and dry sediment weights divided by the sediment initial weight [52]. Sediment porosity and dry bulk density were calculated using formulas from Avnimelech et al. [53] with a particle density of 2.65 g cm^-3^. We determined granulometric properties (sediment mean grain size; MGS) with a HORIBA Partica LA-950 laser diffraction particle size analyzer (Horiba Ltd, Kyoto, Japan). To prepare samples for analyses of total organic carbon (TOC) and total nitrogen (TN), we dried them for 24 h at 80°C, fumed with 1 M HCl for 24 h, and dried them again for a minimum of 24 h. Finally, approximately 2 mg of sediment samples were weighed into a tin capsule and stored at 80°C until analysed in a Perkin-Elmer 2400 Series II CHN analyzer. We used the carbon to nitrogen (C:N) ratio as a measure of organic matter nutritional quality on a long time scale [54], where lower ratios indicate fresher and higher quality organic matter [18, 55].

### Chlorophyll-a and Phaeopigments

Concentrations of chlorophyll-*a* (chl *a*) and phaeopigments (phaeo) were quantified fluorimetrically following a modified version of Riaux-Gobin & Klein [56]. We incubated 1–2 g of wet sediment for 24 h in 90% acetone (v/v) at 4°C and then analysed the supernatant prior to and following acidification using a Turner Designs 10-AU-005-CE fluorometer (Turner Designs, Sunnyvale, USA). The remaining sediment was dried at 60°C for 24 h and weighed in order to standardize pigment concentrations per gram of sediment. The chl *a*:phaeo ratio provides a measure of organic matter quality on a short time scale [54], where higher ratios indicate more recently settled phytoplankton particles and therefore fresher organic matter [57, 58].

### Statistical analyses

We examined spatial and temporal variation in benthic fluxes using a permutational multivariate analysis of variance (PERMANOVA) performed with 9999 random permutations of appropriate units [59, 60]. Three analyses based on subsets of our data addressed three research questions: 1) a one-way PERMANOVA design using all data collected in July 2011 with the factor “Region” (two levels: Salish Sea, NE Pacific) tested regional variability between the Salish Sea and the NE Pacific shelf and slope, 2) a two-way crossed PERMANOVA design tested spatial and temporal variation in the Salish Sea using all data collected in this region with the factors “Date” (three levels: May 2011, July 2011, September 2013), crossed with “Sites” (four levels: SI, SoGC, SoGE, DDL) and their interactions, and finally 3) a one-way PERMANOVA design using data from this region collected in July 2011 with the factor “Sites” (five levels: Axis, BMC, BUP, Folger, Hydrates) tested spatial variation in the NE Pacific. We calculated the resemblance matrix from Euclidean distances of standardized benthic flux and verified homogeneity of multivariate dispersions using the PERMDISP routine [61]. When too few possible permutations were possible to obtain a reasonable test, we calculated a *p*-value based on 9999 Monte Carlo draws from the asymptotic permutation distribution [62]. We further analysed significant terms within the full models using appropriate pair-wise comparisons. We completed PERMANOVA and PERMDISP analyses in PRIMER 6 [63] with the PERMANOVA+ add-on [61].

We determined the model that best explained variation of each benthic flux separately based on environmental drivers using multiple linear regression in the software package R 3.1.1 [64]. Predictor variables containing outliers were transformed, excluding highly correlated (r > 0.95) predictor variables from the analyses. We further analysed multi-collinearity of the predictor variables from the full models with a variance inflation factor (VIF) test using the “vif” function from the “car” package [65], removing predictor variables with the highest VIF so that the best model selected contained only predictor variables with VIF < 5 [66]. Temperature, DO, OPD, chl *a*:phaeo (log_10_), C:N (log_10_), porosity, MGS, and prokaryotic cell abundance (log_10_) were entered into the model as predictor variables. We used Akaike’s information criterion (AIC) to determine the environmental variables best explaining each benthic flux [67], visually verifying residual normality and homogeneity. Because ammonium residual distribution was skewed, we applied a log_10_ transformation to resolve the issue.

We also performed a distance-based redundancy analysis (dbRDA) using the distance-based linear model (distLM) routine from the software PRIMER 6 [63] and the PERMANOVA+ add-on [61]. This ordination technique provided a global understanding of environmental drivers of organic matter remineralization on the seafloor at study locations by analysing all benthic fluxes simultaneously within the same analysis. We determined the model with environmental drivers that best explained variation in benthic fluxes using a stepwise routine that employed 9999 permutations based on AICc selection criterion. This criterion is more appropriate to use with a small (*N*/*v* < 40) ratio of number of samples (*N*) to number of predictor variables (*v*)[61]. Draftsman’s plots of predictor variables indicated high correlation (r > 0.95) between five of our predictor variables (surface sediment phaeo, TN, water content, bulk density and prokaryotic cells biomass) and other predictors; we therefore excluded these five variables from the analysis. The optimal model selection included 13 predictor variables: bottom water temperature, salinity, depth, DO, OPD, surface sediment chl *a*, chl *a*:phaeo ratio, TOM, TOC, C:N ratio, porosity, MGS and prokaryotic cell abundance. To correct for data skewness, we applied a natural logarithmic (Ln) transformation to four predictor variables (chl *a*, chl *a*:phaeo, C:N and Prokabun) and to the response variable O_2_ uptake; the response variable silicate required square root transformation [61]. Prior to distLM, we standardised flux and environmental data using the “normalise” function in PRIMER-E [63]. After standardisation, we created resemblance matrices based on Euclidean distances. We further analysed the multi-collinearity of the predictor variables from the best model with a VIF test as described above, also in R [64], removing predictor variables with a VIF > 5 (i.e. Depth, Sal and chl *a*) prior to selection of the best model [66].

We analysed the effect of air bubbling (two levels: with bubbling, without bubbling) on each benthic flux (O_2_, NH_4_^+^, NO_3_^-^, NO_2_^-^, Si(OH)_4_ & PO_4_^3-^) separately using one-way analysis of variance (ANOVA), verifying normality of residuals and homogeneity of variance visually [67]. We also used PERMANOVA to investigate the effect of air bubbling on all benthic fluxes, following the procedure described above except the PERMANOVA design included only “air bubbling” as a factor (two levels: ambient, oxygenated).

## Results

### Variation of individual benthic flux

**Fig 2:**
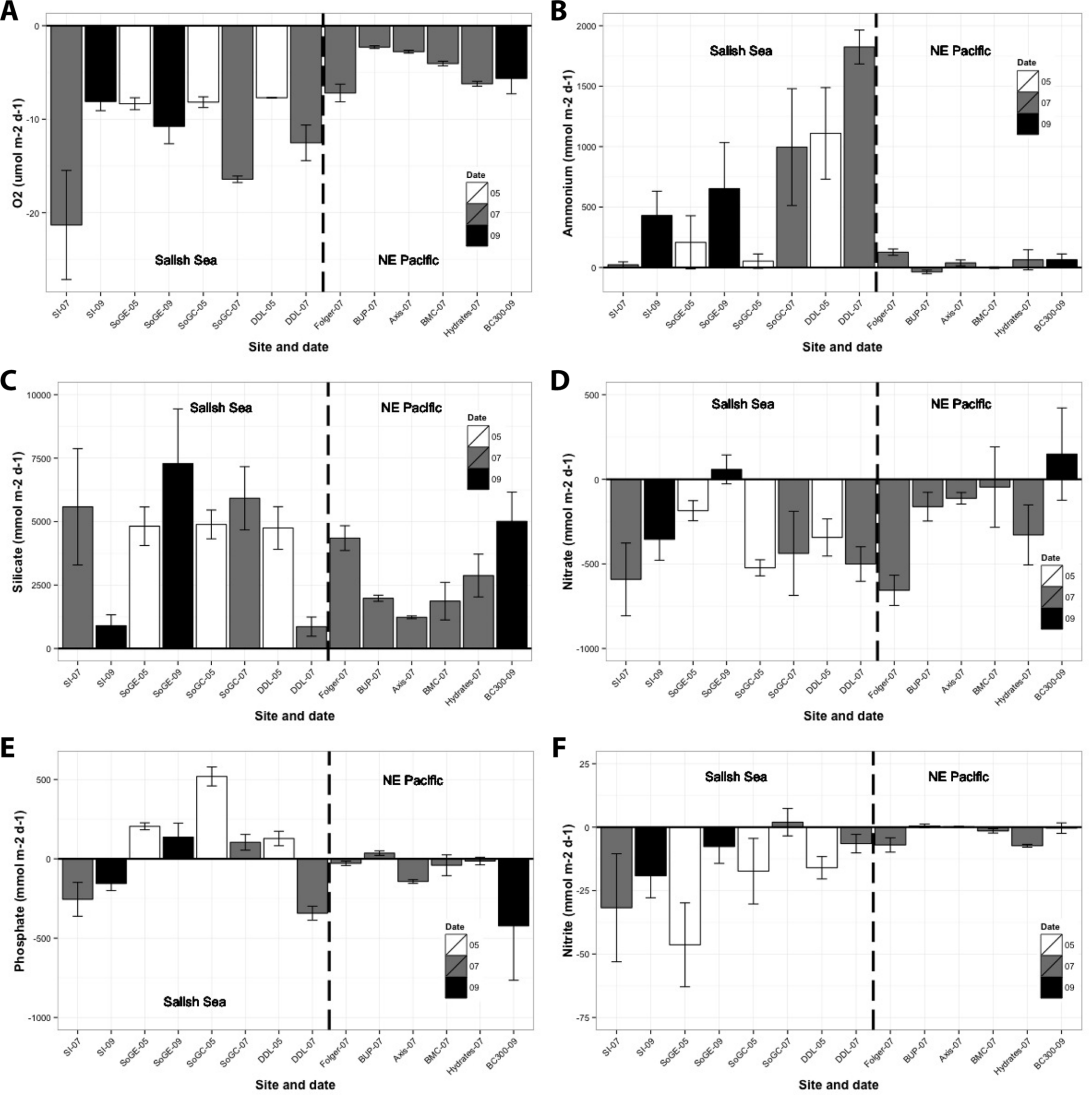
Benthic fluxes (±SE) of a) oxygen, b) ammonium, c) silicate, d) nitrate, e) phosphate and f) nitrite measured at each location. Oxygen uptake is reported in mmol m^-2^ d^-1^ whereas other fluxes units are reported in μmol m^-2^ d^-1^. White bars represent fluxes measured in May 2011, grey bars represent fluxes measured in July 2011, and black bars represent fluxes measured in September 2013. Horizontal lines indicate sediment–water interface where fluxes above the lines represent sediment release and fluxes below the lines represent sediment uptake. Vertical dashed lines separate Salish Sea (left) and NE Pacific (right) stations.

In general, benthic fluxes in the Salish Sea exceeded those in the NE Pacific (Fig 2A–2F, S1 Appendix). Oxygen uptake varied between -2.0 mmol O_2_ m^-2^ d^-1^ in BUP-07 and -17.1 mmol O_2_ m^-2^ d^-1^ in SoGC-07, with one extreme measurement of -32.9 mmol O_2_ m^-2^ d^-1^ in SI-07 (Fig 2A, S1 Appendix). The sediment biota generally released rather than consumed ammonium, and fluxes varied between modest sediment uptakes of -65.9 μmol m^-2^ d^-1^ for BUP-07 to releases of 2202.0 μmol m^-2^ d^-1^ in DDL-07 (Fig 2B, S1 Appendix). The sedimentary biota generally consumed nitrate (except SoGE-09 and BC300-09) with the highest uptake of -1018.6 μmol m^-2^ d^-1^ in SI-07 and highest release of 693.5 μmol m^-2^ d^-1^ in BC300-09 (Fig 2D, S1 Appendix). We observed no clear difference in nitrate fluxes between the Salish Sea and NE Pacific sites. The sedimentary biota also generally consumed nitrite (except SoGC-07 and BUP-07) and fluxes were generally small relative to nitrate flux. We measured the highest uptake of -80.0 μmol m^-2^ d^-1^ in SoGE-05 (Fig 2F, S1 Appendix). Silicate releases from the sediment varied between 130.3 and 13,458.7 μmol m^-2^ d^-1^ in DDL-07 and SoGE-09 respectively (Fig 2C, S1 Appendix). Sediments generally released phosphate in the Salish Sea (except in SI and DDL-07) in contrast to uptake in the NE Pacific (except BUP-07), with highest uptake (-955.4 μmol m^-2^ d^-1^) at BC300-09 and highest release (697.0 μmol m^-2^ d^-1^) at SoGC-05 (Fig 2E, S1 Appendix).

### Regional variation in benthic fluxes

July 2011 offered the only opportunity to compare benthic fluxes directly between the Salish Sea and NE Pacific regions at the same time. PERMANOVA revealed significant differences in multivariate benthic fluxes between the two regions (*P* (perm) < 0.01, Table 2).

**Table 2:** Permutational analysis of variance (PERMANOVA) results testing the effect of sampling date and location on benthic fluxes based on Euclidean similarity matrices performed on normalized data.

**Regional variation between Salish Sea and NE Pacific**				
**Source of variation**	**df**	**MS**	**Pseudo-*F***	***P* (perm)**
Region	1	36.868	7.915	<0.01
Residuals	23	4.658		
Total	24			
**Temporal and spatial variation in Salish Sea**				
**Source of variation**	**df**	**MS**	**Pseudo-*F***	***P* (perm)**
Site	3	13.395	4.092	<0.01
Date	2	15.854	4.843	<0.01
Site x Date	2	5.564	1.700	0.076
Residuals	19	3.273		
Total	26			
**Spatial variation in NE Pacific**				
**Source of variation**	**df**	**MS**	**Pseudo-*F***	***P* (perm)**
Site	4	14.319	4.813	<0.01
Residuals	11	2.975		
Total	15			

### NE Pacific spatial variation in multivariate benthic fluxes

All NE Pacific benthic flux measurements occurred in July 2011, except for flux measurements at BC300 in September 2013. Therefore, we limited spatial analysis of benthic fluxes in this region to July 2011 samples (Deep Barkley Canyon: Axis, Hydrates and BMC; Upper Slope and shelf: BUP and Folger). PERMANOVA analysis indicated significant between site differences (*P* (perm) < 0.01, Table 2). Pair-wise comparisons showed significant differences between Axis and Hydrates (*P* (MC) = 0.026), BUP and Axis (*P* (MC) < 0.01), BUP and Folger (*P* (MC) < 0.01), and BUP and Hydrates (*P* (MC) = 0.042). Folger and Axis (*P* (MC) < 0.01), and Folger and BMC (*P* (MC) = 0.018) also differed significantly.

### Salish Sea spatial variation in multivariate benthic fluxes

Although PERMANOVA analysis of Salish Sea sites sampled during the same time period indicated significant spatial differences (*P* (perm) < 0.01, Table 2), pair-wise comparison tests of benthic fluxes generally showed weak or no significant differences. The strongest flux difference occurred between SI and SoGE in September 2013 (*P* (MC) = 0.030). We observed weak, but significant differences in benthic fluxes in May 2011 between SoGE and SoGC (*P* (MC) = 0.046), and between SoGC and DDL (*P* (MC) = 0.043). SoGC and DDL (*P* (MC) = 0.049) also differed significantly, though weakly, in July 2011. However, we observed no significant difference in benthic fluxes between SoGE and DDL in May 2011, or between SI and SoGC and SI and DDL in July 2011.

### Temporal variation in multivariate benthic fluxes

PERMANOVA analysis of Salish Sea sites sampled at different times indicated significant within-site temporal differences (*P* (perm) < 0.01, Table 2). Pair-wise comparison tests indicated significant temporal differences at SoGC (May and July 2011; *P* (MC) = 0.028) and DDL (May and July 2011; *P* (MC) = 0.026) but no significant temporal differences at SI (July 2011 and Sep 2013; *P* (MC) = 0.088) or SoGE (May 2011 and Sep 2013; *P* (MC) = 0.105).

### Environmental drivers of multivariate benthic fluxes variation

Our best distance-based linear model (distLM) explained 51.5% of total benthic flux variation and included six environmental variables (Table 3). Bottom water temperature contributed most to the variation (16.3%), followed by chl *a*:phaeo ratio (11.8%), C:N ratio (7.9%), DO (6.3%), MGS (4.9%) and porosity (3.8%) (Table 3). The best model excluded O_2_ penetration depth, TOM, C and prokaryotic abundance.

**Table 3:** Distance-based linear model (DistLM) of benthic fluxes against environmental drivers measured in the Salish Sea and NE Pacific in May/July 2011, and September 2013.

Sequential tests for stepwise model (Adj. *r*^2^ = 0.515)							
Variable	AICc	SS (trace)	Pseudo-*F*	*P*	Prop.	Cumul.	Res.df
**Temp**	77.53	43.89	8.54	<0.01	0.163	0.163	44
**Chla:Phaeo**	72.82	31.87	7.06	<0.01	0.118	0.281	43
**C:N**	69.85	21.46	5.22	<0.01	0.079	0.360	42
**DO**	67.18	18.45	4.90	<0.01	0.68	0.428	41
**MGS**	65.68	13.33	3.78	<0.01	0.049	0.478	40
**Porosity**	65.04	10.15	3.03	0.012	0.038	0.515	39

**Fig 3:**
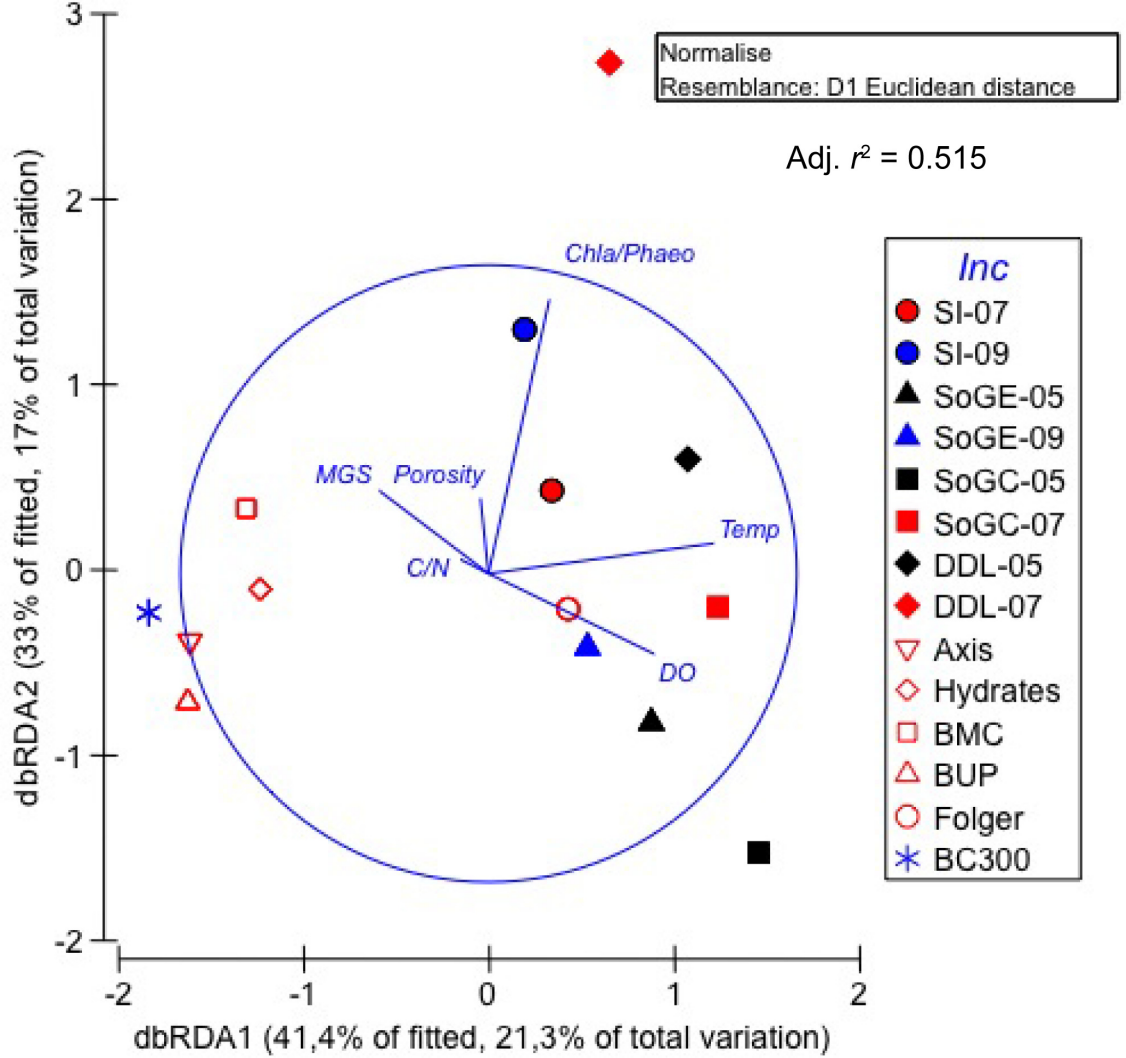
Distance-based Redundancy Analysis (dbRDA) plot of the distLM model of the predictor variables best explaining variation in benthic fluxes measured in the Salish Sea and NE Pacific in 2011 and 2013. Color represents sampling date: Black = May 2011; Red = July 2011; Blue = September 2013. Filled symbols denote benthic fluxes from the Salish Sea and open symbols denote NE Pacific benthic fluxes. Chl *a*:phaeo = Ln of sediment chl *a*:phaeo ratio; C:N = Ln of sediment carbon/nitrogen ratio; DO = bottom water dissolved oxygen concentration; MGS = sediment mean grain size.

The first and second axes of the distance-based redundancy model accounted for 21.3 and 17.0% of total flux variation respectively. The first axis separated Salish Sea and NE Pacific shelf (i.e., Folger) stations from the deeper NE Pacific slope sites (Fig 3). Temp, DO, and MGS contributed primarily to the first axis and explained 41.4% of the fitted fluxes variation (Fig 3, Table 4). Benthic fluxes from the Salish Sea on the second axis varied more than those from the NE Pacific, explaining 33.0% of the fitted variation in fluxes and correlating most strongly with the chl *a*:phaeo ratio (Fig 3, Table 4).

**Table 4:** Percent variation explained by individual axes and relationships between dbRDA coordinate axes and orthonormal variables from Distance-based linear model (DistLM) of benthic fluxes against environmental drivers measured in the Salish Sea and NE Pacific in May/July 2011, and September 2013.

Variation explained by individual axes (%)					Relationships between dbRDA coordinate axes and orthonormal X variables (multiple partial correlations)					
	Explained variation out of fitted model (%)		Explained variation out of total variation (%)		Temp	Chl *a*: Phaeo	C:N	DO	MGS	Porosity
Axis	Ind.	Cumul.	Ind.	Cumul.						
1	41.42	41.42	21.35	21.35	0.732	0.197	-0.091	0.539	-0.355	-0.026
2	33.04	74.47	17.03	38.37	0.098	0.888	0.046	-0.262	0.268	0.244
3	14.47	88.93	7.46	45.83	-0.022	0.069	-0.850	-0.374	-0.349	-0.101
4	7.14	96.07	3.68	49.51	-0.331	0.264	0.415	-0.101	-0.784	-0.152
5	3.87	99.94	2.00	51.50	-0.463	0.313	-0.239	0.580	0.198	-0.506
6	0.06	100	0.03	51.53	0.361	0.008	0.195	-0.394	0.159	-0.807

### Environmental drivers of single benthic flux variation

**Fig 4:**
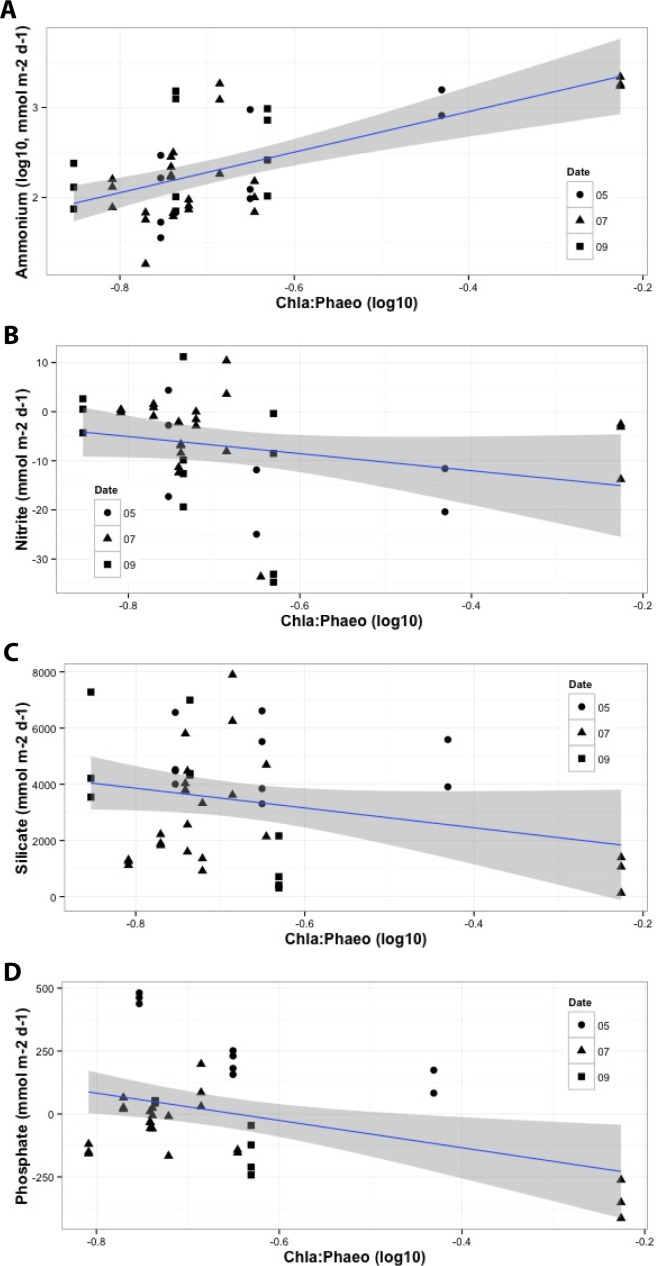
Relationships between sediment chlorophyll *a*:phaeopigment ratio and significant benthic flux of a) ammonium, b) nitrite, c) silicate and d) phosphate identified by multiple linear regression models. Grey shaded area around regression line indicates 95% confidence interval. Sample collection date: May 2011 (circle); July 2011 (triangle); September 2013 (square).

**Fig 5:**
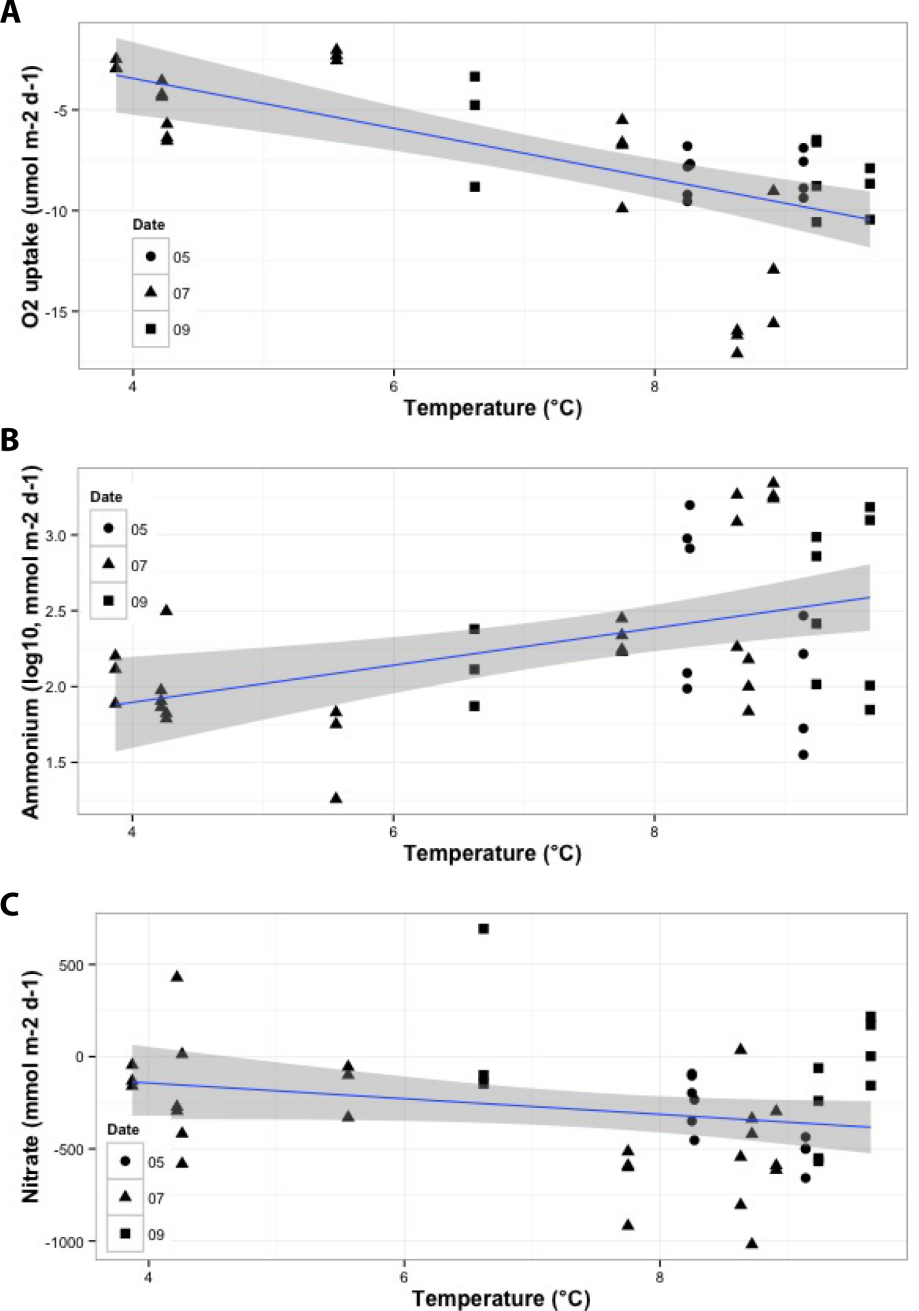
Relationships between bottom water temperature and significant benthic flux of a) oxygen, b) ammonium and c) nitrate identified by multiple linear regression models. Grey shaded area around regression line indicates 95% confidence interval. Sample collection date: May 2011 (circle); July 2011 (triangle); September 2013 (square).

Combinations of the eight primary environmental predictors explained > 50% of the variation in fluxes of oxygen and the five nutrients, except for nitrate and nitrite models, which nonetheless explained 41 and 30% of variance respectively (Table 5). Phosphate flux yielded the best predictive model, increasing with bottom water DO, and decreasing with sediment OPD, chl *a*:phaeo (Fig 4D), porosity, MGS, and prokaryote abundance (Adj. *r*^2^ = 0.88, *p* < 0.001). Oxygen uptake, the second best model, increased with temperature (Fig 5A), OPD, and porosity, and decreased with DO and prokaryote abundance (Adj. *r*^2^ = 0.75, *p* < 0.001). Ammonium flux increased with temperature (Fig 5B), OPD, chl *a*:phaeo (Fig 4A), porosity, and MGS, and decreased with prokaryote abundance (Adj. *r*^2^ = 0.54, *p* < 0.001). Silicate efflux increased with DO and C:N, and decreased with chl *a*:phaeo (Fig 4C) and MGS (Adj. *r*^2^ = 0.51, *p* < 0.001). Nitrate flux increased with DO and C:N, and decreased with temperature (Fig 5C), OPD, and prokaryote abundance (Adj. *r*^2^ = 0.41, *p* < 0.001). Finally, nitrite flux increased with OPD and C:N, and decreased with DO, chl *a*:phaeo (Fig 4B) and prokaryote abundance (Adj. *r*^2^ = 0.30, *p* = 0.003).

**Table 5:** Results of the multiple linear regression models based on AIC. Data utilize are from all sampling locations and dates. DO = Bottom water dissolved oxygen concentration; OPD = Oxygen penetration depth; Chl *a*:Phaeo = Chlorophyll-*a* to phaeopigments ratio; C:N = Carbon-to-nitrogen ratio; MGS = Sediment mean grain size; Prokabun = Prokaryotic cell abundance; RSE = Residuals standard error; * Not significant term in the model but still provides best AIC.

Flux	Intercept	Temp	DO	OPD	Chl *a*: Phaeo (log_10_)	C:N (log_10_)	Porosity	MGS	Prokabun(log_10_)	*r*^2^ (Adj *r*^2^)	*p*-values	RSE
**O_2_**	-44.10	-1.68	0.89	-0.89	N/A	N/A	-0.14	NA	8.01	0.78 (0.75)	<0.001	1.91
**NH_4_^+^ (log_10_)**	3.76*	0.10	N/A	0.04*	1.76	N/A	0.03	0.01	-0.47*	0.60 (0.54)	<0.001	0.37
**NO_3_^-^**	2450.28*	-89.95	90.49*	-33.91*	N/A	703.99	N/A	N/A	-334.13*	0.47 (0.41)	<0.001	253.7
**NO_2_^-^**	20.15*	N/A	-3.88	2.79	-26.52	15.46*	N/A	N/A	-9.72*	0.39 (0.30)	0.003	9.07
**Si(OH)_4_**	-7450.07	N/A	1086.48	N/A	-9272.87	3066.59*	N/A	-11.27*	N/A	0.55 (0.51)	<0.001	1452
**PO_4_^3-^**	1901.99	N/A	97.39	-13.60	-897.19	N/A	-17.27	-5.96	-114.90	0.90 (0.88)	<0.001	69.49

### Effect of overlying water air bubbling on benthic flux rates

Although we took great care to avoid reoxygenating overlying water before incubations, we recorded increases in oxygen concentrations between *in situ* (1.06 mL L^-1^) and *ex situ* conditions (3.83 to 5.12 mL L^-1^) at the beginning of the incubations. Still, PERMANOVA on all benthic fluxes and ANOVAs on separate nutrient fluxes each indicated no significant differences in rates between aerated and non-aerated cores (*P* (MC) = 0.315, O_2_ uptake: *P* = 0.060, ammonium: *P* = 0.455, nitrate: *P* = 0.635, nitrite: *P* = 0.115, silicate: *P* = 0.248, phosphate: *P* = 0.391).

## Discussion

Our study is the first to analyse oxygen and nutrient benthic fluxes along the seafloor affected by the upwelling OMZ waters from the continental slope and shelf off Vancouver Island, to the Strait of Georgia and Saanich Inlet in the Salish Sea. Results demonstrate significant spatial and temporal variation in benthic fluxes resulting from organic matter remineralization, a widely recognized key ecosystem function of benthic habitats [27, 68]. Multivariate statistical analyses (i.e. dbRDA) allowed us to consider environmental drivers of all benthic fluxes simultaneously within the same analysis. Multiple environmental variables drove flux variation, of which, bottom water temperature was most important. Additional major drivers included bottom water DO, quality of organic matter (chl *a*:phaeo and C:N ratios), and sediment characteristics (MGS and porosity).

### Spatial variation

We observed significant spatial variation in flux rates both in the Salish Sea and in the NE Pacific. For NE Pacific sites, benthic fluxes from the continental shelf generally exceeded those from the slope, driven by depth-related environmental drivers. For instance, deeper, colder, and less oxygenated sites from Barkley Canyon within the OMZ, were similar to each other but generally differed significantly from shallower, warmer, and more oxygenated upper slope and shelf sites. Other, more localised environmental drivers such as small-scale variation in sediment chl *a*:phaeo, C:N, MGS and porosity explained smaller differences within Barkley Canyon sites. Relatively large variation in temperature, DO, C:N, porosity, and MGS between shallower NE Pacific sites (BUP and Folger) contributed to between-site spatial differences, keeping in mind depth differences of ~300 m.

The Salish Sea exhibited weak, but significant spatial variation in May and July 2011, in contrast to stronger spatial variation between SI and SoGE in September 2013. These results demonstrate spatial similarity in benthic flux between sites in this region, driven by smaller variation in major environmental drivers compared to those in the NE Pacific. For instance, temperature, the main driver of benthic fluxes in our study, varied by ± 1.38°C over all Salish Sea sampling locations and times, but by ± 3.88°C in the NE Pacific.

### Temporal variation

**Fig 6:**
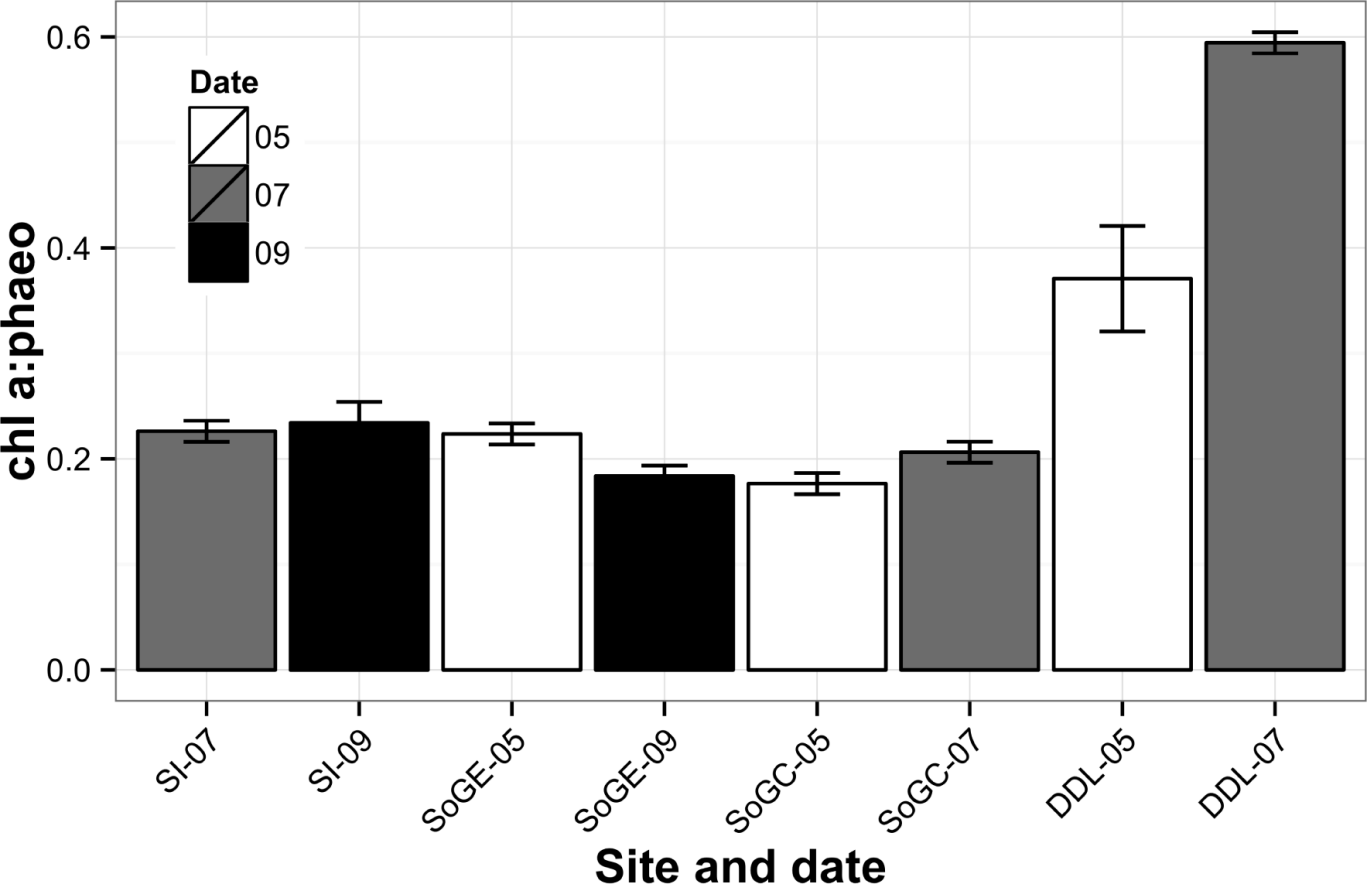
Sediment chlorophyll *a*:phaeopigment ratio (±SE) measured at each location in the Salish Sea. White bars represent fluxes measured in May 2011, grey bars represent fluxes measured in July 2011, and black bars represent fluxes measured in September 2013.

Salish Sea sites differed significantly between May and July 2011 (SoGC and DDL) but not between May 2011 and Sep 2013 (SoGE), and July 2011 and September 2013 (SI). The spring bloom, though variable, generally occurs in April-May in the Strait of Georgia [33] and in Saanich Inlet [69]. Shorter significant blooms occur intermittently over a few days in summer, in contrast to the typically larger fall bloom [33]. The onset of the spring bloom in the Strait of Georgia occurred around April 6–8 in 2011 [70], but settling of the bulk of fresh OM on the seafloor apparently occurred after our measurements in May 2011 and preceded our measurements in July 2011, as shown by increased in chl *a*:phaeo ratios (i.e. short time scale indicator of OM freshness) at DDL between May and July 2011 (Fig 6, Table 1). Increased nutrient fluxes associated with microbial and macrofaunal community responses to fresh organic matter (OM) deposition to the seafloor following the spring and/or summer phytoplankton blooms can therefore explain temporal variations in fluxes at SoGC and DDL between May and July 2011. Similarly, several studies have reported significant increases in oxygen and nutrient fluxes after a spring bloom in San Francisco Bay [71, 72]. Jahnke [25] also linked seasonal variation in benthic fluxes to the reactivity of deposited OM, where seafloor biota quickly metabolized relatively labile OM. In contrast, our study sites exhibited no clear increase in C:N ratios between May and July 2011, arguably because the C:N ratio indicates OM quality on a long time scale [54] and therefore cannot easily indicate fresh OM. The relative stability of other key environmental drivers such as temperature and DO over the two sampling dates in May and July 2011 most likely links to fresh OM deposition on the seafloor following the spring (and possible ephemeral summer) phytoplankton blooms and explains temporal variability, especially at DDL.

### Environmental drivers of individual benthic flux variation

#### Oxygen

Oxygen uptake in the NE Pacific and Salish Sea varied widely, with uptake rates consistent with measurements made on the continental slope and shelf between California and Oregon using benthic chambers [24, 29, 73–76] and eddy-correlation [77], as well as shipboard incubations in the southeast Bering Sea [78].

Previous studies have identified each of the environmental variables highlighted by our multiple linear regression model as major drivers of oxygen uptake. Temperature [79], DO [76], OPD [80], porosity [71], and prokaryotic cell abundance [12, 81, 82] have been found to all influence benthic oxygen uptake. Rowe and Phoel [78] attributed the lack of correlation between sediment oxygen demand (SOD) and depth, temperature, or dissolved oxygen to the small ranges of environmental variables measured. The spatial coverage of our study, which spanned natural environmental gradients, provided an opportunity to measure benthic fluxes over a range of natural variation but within a relatively limited geographic area. This attribute allowed us to identify key environmental drivers of benthic fluxes that may be overlooked in studies that examine a very limited temperature range variation, for example.

**Fig 7:**
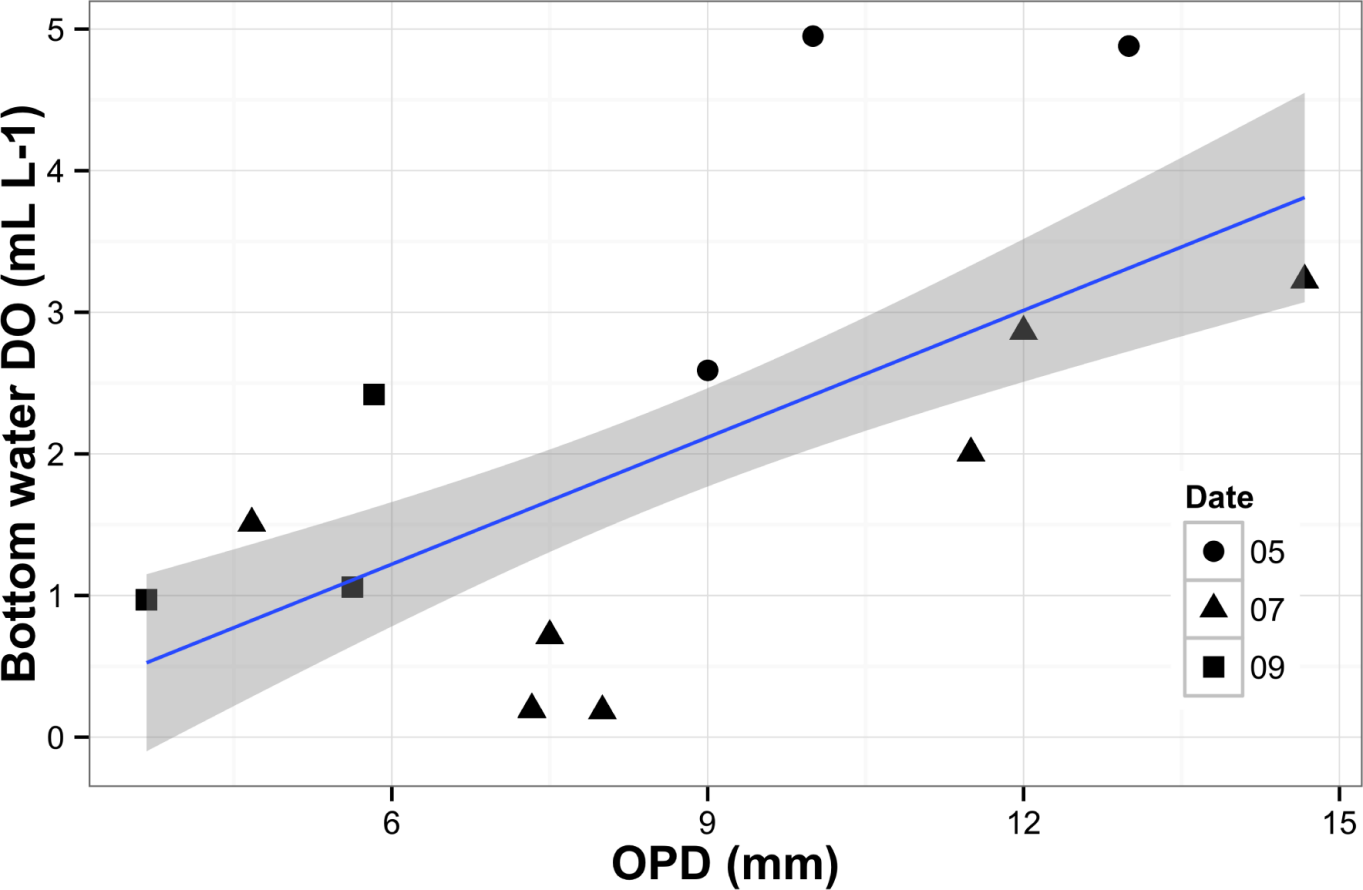
Relationship between oxygen penetration depth (OPD) and bottom water dissolved oxygen (Bottom DO). Grey shaded area around regression line indicates 95% confidence interval. Sample collection date: May 2011 (circles); July 2011 (triangles); September 2013 (squares).

Bio-irrigation may also have influenced oxygen uptake; Archer and Devol [76] found that oxygen uptake estimates using benthic chambers exceeded those measured by microelectrode techniques by 3–4 times. They argued that greater bio-irrigation on shelf sediments could explain this discrepancy because benthic flux calculations based on microelectrode techniques cannot account for macrofaunal irrigation whereas benthic chambers can. The techniques produce similar results in slope sediments because of reduced macrofaunal abundance and bio-irrigation at these depths. We also observed higher macrofaunal abundances at our shelf sites than at our deeper NE Pacific slope sites (R. Belley, unpublished data). Moreover, oxygen penetration, which typically increases in bioturbated and bio-irrigated sediments [83], increased in our study as a function of bottom water oxygen concentration (Fig 7). This increase suggests higher bio-irrigation at the shallower and more oxygenated shelf sites than in deeper and oxygen-depleted slope sites on the NE Pacific.

#### Nitrogen compounds

Given that the marine nitrogen cycle arguably represents the most complex of all biogeochemical cycles in the ocean [84], a complete study of the nitrogen cycle at our sampling stations exceeds the scope of our study. Nonetheless, our analyses indicate important trends in nitrogen cycling within seafloor sediments. Ammonium effluxes were generally low in the Salish Sea before the settling of OM on the seafloor following the spring and smaller summer blooms in May 2011 relative to those measured after the settling of OM on the seafloor following the blooms in July 2011 and September 2013. Whitledge et al. [85] reported a similar increase in ammonium production following the spring phytoplankton bloom in the southeast Bering Sea and subsequent phytoplankton decomposition. Results from our multiple linear regression models indicate prokaryotic cell abundance, a proxy for the primary metabolic driver of benthic biogeochemical processes, influenced patterns for all nitrogen compounds. On the one hand, the overall higher release of ammonium compared to nitrate and nitrite uptake suggest ongoing nitrification and associated chemoautotrophy and ammonium oxidation in the Salish Sea as Whitledge et al. [85] proposed for the Bering Sea. On the other hand, nitrate uptake dominated the NE Pacific, with low ammonium and nitrite fluxes except for BC 300. This pattern suggests ongoing denitrification in our NE Pacific sites. Previous studies emphasize the importance of denitrification as an organic matter oxidation pathway at low bottom water oxygen concentration [86], such as in the OMZ off the coast of Vancouver Island in the NE Pacific.

#### Silicate

Our study shows generally higher silicate effluxes from shallower shelf sites than deeper slope sites, with some seasonal variation. Silicate variation often reflects small-scale differences in sediment properties, bioturbation, and irrigation [78]. Hammond et al. [72] reported a 3–10 fold increase in silica effluxes associated with high phytoplankton productivity and bio-irrigation. Accordingly, our multiple linear regression model identified quality of OM (chl *a*:phaeo and C:N), bottom water DO, and sediment MGS as the best predictors of silicate benthic efflux variation. Because we did not measure bio-irrigation, we cannot directly link silicate effluxes with this variable. However, increased oxygen penetration with bottom water oxygen concentration suggests higher bio-irrigation at the shallower and more oxygenated shelf sites than in deeper and oxygen-depleted slope sites on the NE Pacific. Lower oxygen concentration can also change macrobenthic community structure to one that primarily inhabits and reworks the surface of the seafloor and poorly bio-irrigates sediment at depth [87]. Much higher macrofaunal abundances at the shelf sites than in slope sites (R. Belley, pers. obs.) presumably increased bio-irrigation rates and silicate effluxes [88]. Moreover, Katz et al. [89] suggested that groundfish sediment resuspension in Saanich Inlet triples the flux of dissolved silica from the sediment to the water column and therefore plays a major role in the silica cycle. Both megafaunal and macrofaunal abundance generally decrease with decreasing bottom water dissolved oxygen concentrations [90]. We therefore expect higher silicate effluxes in regions with higher bottom water dissolved oxygen concentrations, given anticipated higher densities of megafauna and macrofauna. Our results support this hypothesis, given that our multiple linear regression model identified bottom water DO as one of the strongest explanatory variables for silicate efflux variation.

#### Phosphate

Many factors influence seafloor phosphate fluxes [91], which vary widely from sediment uptake (-955.4 μmol m^-2^ d^-1^ in BC300) to release (697.0 μmol m^-2^ d^-1^ in SoGC-05). Although Nixon et al. [92] reported increased phosphate fluxes in sediment cores collected before and after the spring bloom in Narragansett Bay, they found no clear seasonal variation. Similarly, Berelson et al. [29] reported little seasonal variation in phosphate fluxes in Monterey Bay shelf sediments. The lack of a seasonal signal in seafloor phosphate flux suggests many factors act on different time scales that no single variable or few variables can explain. Yet, our results indicate that bottom water DO, sediment OPD, chl *a*:phaeo, porosity, MGS and prokaryote abundance can explain 88% of the variation in phosphate flux. Our study apparently encompassed most of the key drivers for phosphate flux, allowing us to predict benthic phosphate flux at these locations. These results are supported by a recent study that modelled benthic phosphate fluxes using three of the variables that we identified (OPD, bottom water DO, and porosity) along with mineral bound inorganic phosphorous [93].

### Environmental drivers of multivariate benthic fluxes variation

Multivariate dbRDA allowed us to examine all benthic fluxes simultaneously within the same analysis. Environmental drivers related to bottom water characteristics, quality of organic matter, and sediment characteristics, explained 51.5% of the variability in overall oxygen and nutrient fluxes. Bottom water characteristics explained most of the variability (temperature and DO, 16.3 and 6.8% respectively), followed by quality of organic matter (chl *a*:phaeo and C:N ratios, 11.8 and 7.9% respectively) and sediment characteristics (MGS and porosity, 4.9 and 3.8% respectively). In their Beaufort Sea study, Link et al. [30] also reported upper sediment concentrations of chl *a* and phaeopigments, and bottom water dissolved oxygen as key environmental drivers of multivariate benthic flux variation. However, in contrast to our study, where temperatures varied by >5.5°C across sampling locations and dates, temperatures recorded at the time of sampling varied by <2°C and therefore contributed little to benthic flux variation, just as Rowe and Phoel [78] suggested for the Bering Sea. Because temperature increase promotes bacterial production [94], which in turn influences rates of benthic processes, increased benthic fluxes could reasonably be expected along a natural gradient of increasing temperature.

The dbRDA and multiple linear regression approaches identified some differences in environmental drivers of benthic fluxes. On the one hand, the dbRDA approach combines all benthic fluxes (O_2_ uptake, ammonium, nitrate, nitrite, silicate and phosphate) in the same analysis to determine the best combination of environmental drivers for all benthic fluxes, hence of seafloor organic matter remineralization. On the other hand, the multiple linear regression approach analysed each benthic flux separately (e.g., ammonium) to determine the best combination of environmental drivers for each specific benthic flux. Given some commonalities in benthic fluxes but different degrees of influence by some environmental drivers on each benthic flux (e.g., temperature influences O_2_ uptake but not phosphate release), the linear regression method identifies the environmental drivers of each benthic flux whereas the dbRDA method identifies the common environmental drivers that influence all benthic fluxes and examines seafloor organic matter remineralization as a whole.

The environmental variables we measured could not explain approximately 48.5% of the variability in benthic fluxes. Therefore, biological and environmental factors not measured in this study could also contribute to benthic flux variation. Additional factors known to influence benthic flux rates include sediment resuspension by megafauna [95], bio-irrigation [74, 76, 88], bacterial activity [12] and production [82], meiofaunal abundance [96, 97], macrofaunal abundance [78] and species richness [18], functional diversity [88] and particulate organic carbon flux to the seafloor [24, 25]. Measurement of some of these parameters in tandem with those reported here would likely increase the capacity of future studies to explain benthic flux variation more fully.

### Effect of overlying water air bubbling on benthic flux rates

Our complementary experiment revealed no significant effect of air bubbling of the overlying water on any of our benthic flux rate measurements. These results corroborate previous incubation studies that found no significant effect of incubation time (i.e., oxygen concentration changes within incubations) on flux rates of oxygen, nitrate, phosphate, silicate and, for the most part, ammonium, where flux rates changed only after oxygen decreased by 50–80% from ambient values [73, 74]. This insensitivity demonstrates the absence of a short-term response in flux of these compounds to changes in oxygen concentration and, therefore, to oxygen concentrations at the onset of incubations. Consequently, we believe our benthic flux measurements represent realistic estimates of ambient benthic flux rates at our study sites.

## Conclusions

Our study indicates strong variation in spatial and temporal flux, driven primarily by differences in bottom water characteristics (bottom water DO and temperature), quality of organic matter (chl *a*:phaeo and C:N ratios) following significant deposition of particulate organic matter to the seafloor and, to a lesser extent, sediment characteristics (MGS and porosity). Although multiple biological and environmental factors influence different seafloor flux rates (O_2_ and nutrients), our study used a suite of multivariate approaches in tandem to demonstrate that a subset (i.e., 6) of the large number of environmental variables measured (i.e., 18) could explain benthic flux variation. We also found that simultaneous and single flux analyses in tandem provided a more comprehensive understanding of the interplay between OM remineralization and flux of O_2_ and individual nutrients.

The large variation in natural gradients (e.g., bottom water temperature and dissolved oxygen concentration) at our study sites allowed us to identify bottom water temperature as the key driver of benthic flux variation. These results indicate that current and future predictive models of organic matter remineralization and ecosystem functioning of shelf and slope soft-muddy seafloor habitats should consider bottom water temperature variation. Temperature could have important implications for estimates of seasonal and spatial benthic flux variation, benthic-pelagic coupling, and potential impacts of predicted ocean warming, particularly at high latitudes.

## Supporting Information

S1 Appendix Benthic fluxes measured in the Salish Sea and NE Pacific in May/July 2011, and September 2013. (DOCX)
