# Supplementary material for: Environmental Drivers of Benthic Flux Variation and Ecosystem Functioning in Salish Sea and Northeast Pacific Sediments
Source: PLoS One. 2016 Mar 4;11(3):e0151110. doi: 10.1371/journal.pone.0151110 (PMC4778862; doi:10.1371/journal.pone.0151110)
Supplement: S1 Appendix — (DOCX) [file pone.0151110.s001.docx]

S1 Appendix: Benthic fluxes measured in the Salish Sea and NE Pacific in May/July 2011, and September 2013.

| **Station/**  **label** | **O_2_ uptake**  **(mmol/m^2^/d)** | **Ammonium**  **(μmol/m^2^/d)** | **Nitrite**  **(μmol/m^2^/d)** | **Nitrate**  **(μmol/m^2^/d)** | **Silicate**  **(μmol/m^2^/d)** | **Phosphate**  **(μmol/m^2^/d)** |
| --- | --- | --- | --- | --- | --- | --- |
| SI-1-07-11 | -14.10 | 15.78 | 6.02 | -336.43 | 2138.52 | -142.78 |
| SI-2-07-11 | -16.97 | -15.54 | -33.62 | -419.61 | 4688.91 | -154.09 |
| SI-3-07-11 | -32.86 | 67.08 | -67.65 | -1018.62 | 9924.88 | -468.45 |
| SI-6-09-13 | -8.78 | 885.46 | -8.50 | -550.91 | 413.41 | -122.91 |
| SI-7-09-13 | -6.49 | 19.54 | -34.70 | -567.28 | 710.14 | -242.30 |
| SI-8-09-13 | -10.57 | 639.34 | -0.39 | -239.79 | 2162.96 | -45.51 |
| SI-10-09-13 | -6.61 | 176.10 | -33.08 | -61.88 | 307.42 | -210.98 |
| SoGE-3-05-11 | -9.54 | 863.11 | -68.70 | -349.43 | 6610.73 | 156.72 |
| SoGE-4-05-11 | -7.82 | 38.87 | -11.85 | -92.38 | 3844.44 | 181.41 |
| SoGE-5-05-11 | -6.80 | -83.22 | -24.95 | -103.73 | 3302.75 | 251.75 |
| SoGE-6-05-11 | -9.21 | 12.91 | -79.96 | -196.79 | 5515.63 | 230.41 |
| SoGE-16-09-13 | -16.08 | 1440.86 | -12.62 | 2.69 | 6995.57 | 51.93 |
| SoGE-17-09-13 | -10.45 | -13.68 | -19.39 | 170.92 | 4322.76 | 42.63 |
| SoGE-18-09-13 | -8.66 | 17.64 | -9.86 | -157.02 | 4381.93 | 53.20 |
| SoGE-20-09-13 | -7.90 | 1167.56 | 11.17 | 217.72 | 13458.67 | 400.82 |
| SoGC-7-05-11 | -8.89 | 209.37 | -2.78 | -658.15 | 6556.52 | 438.03 |
| SoGC-8-05-11 | -7.56 | -31.04 | -17.28 | -497.88 | 4002.87 | 481.13 |
| SoGC-9-05-11 | -9.37 | 80.12 | 4.36 | -500.60 | 4521.22 | 696.98 |
| SoGC-10-05-11 | -6.89 | -48.46 | -53.70 | -435.92 | 4473.27 | 462.30 |
| SoGC-4-07-11 | -16.19 | 1134.08 | -8.10 | -543.99 | 6247.31 | 29.88 |
| SoGC-5-07-11 | -17.11 | 98.11 | 3.56 | 35.63 | 3617.25 | 85.26 |
| SoGC-6-07-11 | -15.97 | 1755.44 | 10.36 | -804.99 | 7895.01 | 198.26 |
| DDL-11-05-11 | -7.73 | 730.64 | -20.40 | -233.74 | 3908.53 | 82.82 |
| DDL-12-05-11 | -7.68 | 1488.70 | -11.60 | -453.33 | 5588.30 | 174.08 |
| DDL-7-07-11 | -15.60 | 2102.01 | -2.56 | -590.94 | 1394.34 | -351.40 |
| DDL-8-07-11 | -12.94 | 1649.75 | -13.78 | -614.12 | 130.33 | -414.38 |
| DDL-9-07-11 | -9.03 | 1720.78 | -3.13 | -296.81 | 1059.78 | -262.00 |
| Axis-15-07-11 | -2.94 | 46.42 | 0.32 | -158.99 | 1120.08 | -119.46 |
| Axis-16-07-11 | -2.48 | -6.90 | 0.36 | -45.32 | 1260.36 | -151.10 |
| Axis-17-07-11 | -2.92 | 74.92 | -0.18 | -131.82 | 1306.47 | -157.52 |
| Hydrates-29-07-11 | -6.54 | -22.60 | -6.90 | -417.79 | 2557.70 | -6.07 |
| Hydrates-30-07-11 | -5.70 | -17.31 | -6.66 | 13.09 | 4470.22 | 23.43 |
| Hydrates-31-07-11 | -6.39 | 229.95 | -8.42 | -581.40 | 1597.47 | -58.38 |
| BMC-18-07-11 | -4.36 | -3.54 | -0.02 | -294.73 | 918.12 | -166.59 |
| BMC-19-07-11 | -4.23 | 10.36 | -1.58 | 429.28 | 3322.99 | 54.61 |
| BMC-20-07-11 | -3.55 | -10.81 | -2.89 | -272.19 | 1357.28 | -9.01 |
| BUP-21-07-11 | -2.03 | -65.87 | -0.93 | -99.57 | 1825.42 | 19.01 |
| BUP-22-07-11 | -2.55 | -27.61 | 0.84 | -329.61 | 1895.60 | 25.09 |
| BUP-23-07-11 | -2.30 | -16.43 | 1.51 | -55.88 | 2217.23 | 64.86 |
| Folger-24-07-11 | -5.51 | 84.54 | -2.12 | -591.16 | 3779.39 | -31.81 |
| Folger-25-07-11 | -6.63 | 91.27 | -2.23 | -600.24 | 5801.80 | -33.45 |
| Folger-26-07-11 | -6.74 | 133.86 | -11.33 | -918.16 | 4028.47 | -56.87 |
| Folger-27-07-11 | -9.90 | 197.34 | -12.42 | -514.60 | 3790.46 | 10.17 |
| BC300-11-09-13 | -4.76 | 155.33 | 0.51 | 693.46 | 7280.37 | 217.00 |
| BC300-12-09-13 | -8.82 | 45.80 | 2.62 | -146.79 | 3542.03 | -955.35 |
| BC300-13-09-13 | -3.34 | -9.68 | -4.32 | -99.64 | 4212.73 | -529.36 |
